# Comparison of Radiomic Features from Different MRI Sequences for Predicting Synchronous Liver Metastases after Rectal Cancer

**DOI:** 10.2174/0115734056399652251029204023

**Published:** 2025-11-28

**Authors:** Apekshya Singh, Sheng-Ming Shi, Han Liu, Yu-peng Wu, Yuhang Wang, Jiayi Xie, Xiao-Fu Li

**Affiliations:** 1 Department of Magnetic Resonance Imaging, The Second Affiliated Hospital, Harbin Medical University, Harbin 150086, Heilongjiang Province, China

**Keywords:** Synchronous liver metastasis, Rectal cancer, Radiomics, Random forest, Machine learning, T2-weighted imaging, Diffusion-weighted imaging, Predictive model, LASSO, ITK-SNAP

## Abstract

**Introduction:**

Synchronous liver metastases (SLM) critically influence prognosis in rectal cancer, highlighting the need for accurate preoperative detection. This study aimed to compare the predictive performance of radiomic features extracted from T2-weighted imaging (T2WI) and diffusion-weighted imaging (DWI) MRI sequences and to develop machine learning-based predictive models for the early detection of SLM in rectal cancer patients.

**Methods:**

This retrospective study included 160 rectal cancer patients confirmed by pathology at our institution between September 2018 and June 2023. After screening, 137 patients were enrolled, comprising 71 patients with SLM and 66 without SLM. Clinical characteristics such as age, gender, tumor (mrT) staging, lymph node (mrN) staging, tumor size, tumor distance from the anal verge, location, and circumferential range were analyzed, with mrT and mrN staging showing statistical significance (*p* < 0.012). Radiomic features were extracted from regions of interest (ROIs) on T2WI and DWI using Pyradiomics after manual segmentation in ITK-SNAP. A total of 3,452 radiomic features (1,726 each from T2WI and DWI) were extracted, of which 14 features (4 from T2WI and 10 from DWI) were selected using the LASSO. Predictive models were developed using three machine learning algorithms: Logistic Regression (LR), Support Vector Machine (SVM), and Random Forest (RF), with a five-fold cross-validation strategy.

**Results:**

Among the machine learning algorithms, the RF consistently outperformed LR and SVM across all models. The Optimal model yielded the highest predictive performance, with RF achieving an AUC of 0.82 (95% CI: 0.66–0.93), an accuracy of 0.71, and an F1-score of 0.74. RF also showed superior performance in the Combined-Optimal model (AUC = 0.76, accuracy = 0.71). In contrast, models built using LR and SVM algorithms demonstrated moderate performance, with lower AUC values ranging from 0.68 to 0.70. Confusion matrix analysis confirmed RF’s superior classification ability, accurately predicting SLM and non-SLM cases.

**Discussion:**

The incorporation of radiomics and RF-based models conveys a promising, non-invasive approach for enhancing early detection and risk stratification of SLM, which could help with more reliable clinical decision-making and individualized treatment planning for patients with rectal cancer.

**Conclusion:**

The optimal feature set-based predictive model demonstrated the highest accuracy for SLM detection, with the RF algorithm outperforming LR and SVM by consistently achieving the best AUC and balanced diagnostic performance.

## 1 INTRODUCTION

Rectal cancer is a highly prevalent malignancy with significant morbidity and mortality, affecting primarily middle-aged and older adults, though an increasing incidence among patients of younger ages has been observed in recent years [1]. Globally, it accounts for over 1 million deaths on an annual basis [1]. Tumor metastatic spread and local recurrence are both primary causes of death among individuals with rectal cancer. About 35–55% of patients with rectal cancer develop liver metastases, being the most frequent target organ for hematogenous metastases of the rectal cancer, and ultimately, 40–50% of these patients pass away as a consequence of liver metastases [2]. Synchronous liver metastasis (SLM) refers to liver metastases detected simultaneously or within a short period after a primary cancer diagnosis. Many studies use the 6-month cutoff for determining treatment choices and prognosis, and it is a commonly utilized practical and operational definition in radiology literature and clinical practice to recognize SLM from metachronous liver metastases (MLM) [3-5]. Major clinical study findings and standards tend to apply this threshold for the reason that it is relevant in capturing early metastatic progression, regardless of certain studies acknowledging that it seems more convention-based than biologically favored. The leading cause of mortality for individuals with rectal cancer (RC) is synchronous liver metastasis (SLM), which exists in 15%–20% of patients at the time of diagnosis [6]. As shown by international studies, patients with synchronous liver metastases have a 1-year survival rate of just 34.8% and a 5-year survival rate of just 3.3% [7]. The prognosis of rectal cancer is strongly influenced by the presence of synchronous liver metastases, which are associated with a poorer outcome compared to metachronous liver metastases [8].

Based on recent research, neoplastic heterogeneity has been correlated to certain tumor metastases [9]. Conventionally, physicians assess tumor characteristics based on the imaging features of lesions. However, this approach mostly depends on the expertise and experience of the clinicians, leading to variations in lesion assessments among individuals and a lack of consistency [10]. Currently, there are limited reliable methods for predicting SLM in rectal cancer, resulting in many patients missing the optimal timeframe for surgical resection by the time liver metastases are detected. Assessing the recurrence and/or metastasis status in rectal cancer patients before surgery is essential for determining appropriate treatment strategies [11]. The recommended practices state that individuals with rectal cancer who have synchronous liver metastases and those who do not have synchronous liver metastases get quite different treatment approaches. Therefore, accurately predicting SLM pre-operatively and noninvasively is essential for developing individualized treatment strategies. However, this remains a significant challenge, making it essential for clinicians to develop reliable methods for accurately identifying the metastasis status of malignancy before initiating treatment. Therefore, to enable tailored clinical therapy and enhance prognosis, an accurate identification approach that may be utilized to more precisely and early predict liver metastasis in rectal cancer patients must be constructed.

CT imaging has limited sensitivity for detecting synchronous liver metastases smaller than 10 mm and can sometimes struggle to differentiate them from other small intrahepatic lesions, such as hemangiomas or cysts. Furthermore, concerns regarding radiation exposure restrict the routine use of CT for identifying synchronous liver metastases [12]. Since MRI is better able to describe the primary tumors, extramural vascular invasion (EMVI), and liver metastases from rectal cancer, it has emerged as the preferred imaging modality for staging the disease [13]. MRI was the recommended first-line method for the preoperative clinical evaluation of SLM because of its high sensitivity and specificity when compared to the other major modalities, particularly for lesions smaller than 1 cm [14]. The second-line strategy is PET-CT [15]. Nevertheless, these imaging modalities' diagnostic accuracy is unsatisfactory [15, 16]. SLM is still not detectable by conventional MRI. Previous research has shown the viability of using clinicopathological characteristics to assess the possible risk of SLM in rectal cancer [17]. However, certain indicators are only obtainable after radical resection, limiting their utility in guiding preoperative treatment plans. Hence, there is a need to develop a preoperative, noninvasive, and precise method for predicting SLM.

In recent times, radiomics has shown encouraging results in rectal cancer monitoring, characterization, and detection [18]. Radiomics is a new area that may convert internal heterogeneity information into high-dimensional quantitative image features and extract many visually unidentifiable imaging features. Radiomics primarily involves high-quality image acquisition, precise ROI delineation, extraction of imaging features, and the establishment and validation of predictive models. Several prior studies have utilized basic radiomics derived from CT images of liver parenchyma for the earlier detection of liver metastases [19, 20]. However, limited research has focused on utilizing radiomic features extracted from MRIs taken from primary rectal cancer to predict SLM. According to certain theories, MRI-radiomics of CRC patients could serve as a non-invasive method of predicting the risk of SLM [21]. According to certain research, radiomics models can forecast distant metastases in a variety of primary malignancies [18, 22]. However, it is still unclear how the radiomics model based on the primary lesion predicts SLM in RC patients. Numerous studies [23] have demonstrated that combining multi-parametric MRI (mp-MRI) sequences provides a comprehensive analysis of lesions. mp-MRI radiomics can more accurately represent the heterogeneity of tumors than single MRI sequence radiomics. Some researchers [24] built predictive radiomic models using mp-MRI, but they lacked external data for confirming the model's stability. Research has highlighted that there is an urgent need to choose higher-order features with reproducibility in radiomics and to improve the overall methodological quality of radiomics studies [25]. Hence, this study aims to compare the predictive performance of radiomic features extracted from T2-weighted imaging (T2WI) and diffusion-weighted imaging (DWI) MRI sequences. Additionally, the study seeks to develop machine learning-based predictive models using these radiomic features for the early detection of synchronous liver metastases (SLM) in patients with rectal cancer.

## 2 METHODS

### 2.1 Sample Population and Research Approach

This research was conducted as a retrospective study and received approval from the Ethics Committee of the 2nd Affiliated Hospital of Harbin Medical University. As all patient information was anonymized, informed consent was not required. The research processes were conducted according to the 1964 Declaration of Helsinki. The data of 160 consecutive patients with histopathologically confirmed rectal cancer at our hospital between September 2018 and June 2023 were retrospectively analyzed. Based on the inclusion and exclusion criteria outlined in Fig. (**1**), a total of 137 patients were selected and divided into two groups. The SLM group (n = 71) included patients diagnosed with SLM within six months of primary rectal cancer diagnosis. In contrast, the non-SLM group (n = 66) consisted of patients with no liver metastasis identified within six months following the diagnosis and treatment of primary rectal cancer. All 71 patients in the SLM group were followed up for at least six months, with the earliest follow-up starting in September 2018 and the latest beginning in December 2022. Both groups' initial diagnostic and follow-up data were complete.

The recorded general characteristics included gender, age, primary rectal cancer location, MRI T and N staging, tumor size, distance of the lesion from the anal verge, and its circumferential extent. All participants underwent rectal MRI to determine the local staging of rectal cancer. The inclusion criteria were as follows: (1) patients with a confirmed pathological diagnosis of rectal cancer; (2) patients who underwent preoperative high-resolution rectal MRI for local staging of rectal cancer; (3) patients who had abdominopelvic CT, ultrasound (USG), or liver MRI to assess liver metastases; (4) MRI images that were clear and interpretable; and (5) complete imaging and follow-up data. The exclusion criteria included the following: (1) patients who received local or systemic treatment before the baseline MRI; (2) cases without a pathological confirmation of rectal cancer; (3) cases with poor-quality imaging; and (4) incomplete clinical, imaging, pathological, or follow-up data. Thus, the final study cohort included 137 patients. The flow diagram outlining patient recruitment is illustrated in Fig. (**1**).

### 2.2 MRI Acquisition Parameters

The patient was instructed to fast for 4 hours before the examination. During the examination, the patient was asked to lie flat on the examination bed, and the scanning position was head first and supine. All patients underwent a pre-treatment MRI scan for staging and feature extraction, using 3.0 T MR scanners (SignaHDx and GE Discovery MR 750, General Electric Medical Systems, Milwaukee, WI) equipped with a phased-array coil at a single center. The sequences chosen for this study were T2WI and DWI sequences. MRI characteristics are distinctive in rectal adenocarcinoma: T2WI shows the tumor with a signal intensity greater than the muscularis propria but less than the submucosa. On high b-value DWI, the lesions exhibit notably higher signal intensity than the normal rectal wall tissue [26].

Scanning parameters are shown in Table **1**. MRI scan sequences in this study include:

(1) High-definition (HD) sagittal T2-weighted image,

(2) HD T2WI (TR: 5990 ms, TE: 125.7 ms, FOV: 200 × 200 mm2, matrix: 320 × 320, slice thickness: 5.0 mm, slice spacing: 6.0 mm) in oblique axial view (perpendicular to the long diameter of the tumor),

(3) Axial fat-suppressed T2WI, and

(4) Axial diffusion-weighted imaging (DWI), (TR: 4881 ms, TE:72 ms, FOV: 280 × 280 mm2, slice thickness: 5.0 mm, slice spacing: 6.0 mm, b value 1000 s/mm2).

### 2.3 Collection of Clinical Features

The clinical features of patients in both the SLM and non-SLM groups were systematically gathered, with non-radiomic MRI features categorized as clinical to avoid confusion with radiomic data. These features included demographic and imaging-based details such as age, gender, MRI-based T and N stages (mrT and mrN), lesion length, distance from the lesion’s lower edge to the anal verge, and the circumferential extent of the lesion (categorized as <1/4, 1/4–1/2, 1/2–3/4, or 3/4–1 of the rectal circumference). The mrT and mrN stages were determined based on the 8^th^ edition of the American Cancer Society’s TNM staging guidelines for rectal cancer [27]. Essential measurements, including tumor length and the distance from the tumor’s lower edge to the anal verge, were extracted from each patient’s preoperative electronic medical records to maintain consistency. An attempt was made to include important non-radiomic factors determining the patient’s profile, staging, and possible prognosis by describing clinical features in this manner. A senior radiologist with over 10 years of expertise in rectal MRI interpretation thoroughly reviewed all the listed features. This meticulous evaluation ensured data reliability and accuracy, ensuring consistency across cases, minimizing variability in feature assessment, and improving the overall quality of data for analysis.

### 2.4 Segmentation of Images

Images in Digital Imaging and Communications in Medicine (DICOM) format were retrieved from our hospital's Picture Archiving and Communication System (PACS). 3D Slicer software (version 5.2) was used to standardize the original DICOM images of 137 patients who had recently been diagnosed with rectal cancer. All MRI images were resampled to a uniform voxel size of 1.0 mm × 1.0 mm × 1.0 mm in the X, Y, and Z dimensions using the ‘Resample Scalar Volume’ module in 3D Slicer to standardize spatial resolution, and z-score intensity normalization was subsequently employed to mitigate inter-scan intensity variability before radiomic feature extraction with PyRadiomics**. **A Gaussian filter was used to smooth out variations in intensity and minimize noise to improve image quality while maintaining crucial anatomical characteristics that were vital to the analysis. The intensity measurements were then normalized to a specified range, which reduced variability caused by various imaging settings and enhanced consistency between scans, thus boosting the robustness of radiomic feature extraction. ITK-SNAP software (Penn Image Computing and Science Laboratory, PICSL, USA) was then used to import these standardized MRI images [28]. For manual segmentation of rectal tumors, ITK-SNAP (version 3.8.0, http://www.itksnap.org/) was utilized.

The images were initially evaluated using axial and sagittal T2WI to define the tumor boundaries and identify the largest cross-sectional area of the lesion. Figure (**2A**-**F**) showcases various colormap visualizations that enhance the tumor's characteristics, while the original grayscale MRI data has been utilized for radiomic feature extraction. Regions of interest (ROIs) were meticulously outlined around the entire tumor in the largest cross-section of both T2WI and DWI (b = 1000) images. The process involved: (a) delineating the ROI on the tumor's maximum cross-section in oblique axial T2WI; and (b) using the T2WI ROI to guide the delineation of the corresponding ROI in the DWI image, as shown in Fig. (**3A**-**F**). To ensure precision, each ROI was then reviewed, verified, and, if necessary, modified by a senior radiologist with extensive expertise in MRI-based tumor delineation. To ensure consistency as well as reliability in tumor segmentation, the senior observer completed the margins in cases where the difference between the initial and revised ROIs was ≥5%. In order to preserve accuracy across patient datasets and ensure that the subsequent radiomic analyses depend on reliable, consistent segmentations, this standardized approach was needed. Additionally, radiologists have been blinded to clinical and pathological findings, ensuring an unbiased imaging assessment.

### 2.5 Extracting and Selecting Features

One essential and crucial role in the processing of medical images is feature extraction. The goal is to derive feature vectors, symbols, and numerical values that represent the characteristics of samples. Classification accuracy is significantly impacted by the feature extraction that results from it. In this study, radiomic features were extracted from regions of interest (ROIs) on T2WI and DWI using Pyradiomics. Following image preprocessing to ensure consistency and quality, high-throughput data features were automatically extracted from the platform using the “pyradiomics” package in Python (version 2.1.2, https://pyradiomics.readthedocs.io/). PyRadiomics, Numpy, SimpleITK, PyWavelet, and Python were the versions of the dependencies used. A total of 3,452 features were extracted, with 1,726 features derived from each imaging modality. The feature breakdown includes 324 features from first-order statistics, 14 features from shape, 432 features from GLCM (Gray-Level Co-occurrence Matrix), 288 features from GLRLM (Gray-Level Run-Length Matrix), 288 features from GLSZM (Gray-Level Size-Zone Matrix), 252 features from GLDM (Gray-Level Dependence Matrix), and 90 features from NGTDM (Neighborhood Gray-Tone Difference Matrix). Additionally, 38 higher-order statistical features were also extracted. Furthermore, significant features were identified using the Mann-Whitney U test, an effective statistical method for comparing two groups, particularly with binary categorical variables (*e.g*., 1 for “yes” and 0 for “no”). Features with a *p*-value less than 0.05 were considered statistically significant. For T2WI, 131 significant features were identified. Similarly, for DWI, 83 significant features were identified. Metrics of intensity, including mean, standard deviation, variance, maximum, median, and range, were quantified throughout first-order statistical features. Geometric properties such as volume, surface area, compactness, and maximum diameter were characterized in shape features. Texture features used matrices like GLCM, GLSZM, GLDM, NGLDM, and GLRLM to record regional heterogeneity differences. Additionally, higher-order statistical features, obtained *via* wavelet transformation, encompassed both first-order and texture features. Eight feature forms, designated LLL, LLH, LHL, LHH, HLL, HLH, HHL, and HHH, are produced by the wavelet transform of decomposed regions of tumor into low- and high-frequency components (L and H) along the x, y, and z axes. Further transformations involving logarithms, exponentials, gradients, squares, square roots, and local binary patterns (LBP) extended the feature set, further increasing heterogeneity analysis. The precise description of radiomics features is included in the **(Figs. **S1**-**S4**)**.

A key phase when building a radiomic model is feature selection, which keeps the model confined to the features that are crucial and improves its interpretability and predictive performance by eliminating redundant features and keeping the important ones. This process additionally assists in preventing overfitting while raising robustness and reproducibility. The most relevant features for predicting SLM were selected using a multi-step feature selection strategy. Firstly, the Mann-Whitney U Test was applied separately to each imaging sequence (T2WI and DWI) to compare each radiomic feature between the SLM and Non-SLM groups. T2WI and DWI were not treated as experimental and control groups; rather, each sequence was analyzed separately to identify features. Only continuous radiomic features were included during this step, while categorical variables such as clinical or demographic data were excluded from the analysis. Secondly, the least absolute shrinkage and selection operator (LASSO) technique. By applying regularization, LASSO efficiently reduces regression coefficients toward zero and assigns zero values to non-contributory features based on the regularization parameter (α). This approach is especially valuable for high-dimensional radiomic data, generating a sparse yet interpretable feature set that retains only the most predictive features. Leave-one-out cross-validation was conducted to determine the optimal regularization parameter (α). In order to balance model complexity and performance, the α value was determined to minimize the mean squared error among all patients. A final rounded α value was applied to further refine feature selection. Additionally, features with coefficients between -0.01 and +0.01 were excluded to ensure that only the most informative features were preserved. Our empirical threshold of ±0.01 serves as a practical refinement of LASSO’s built-in tendency, retaining features with meaningful contributions while filtering out minimal noise. Finally, pairwise correlation analysis with visual inspection (correlation heatmaps) was performed to identify and remove highly collinear features to reduce further redundancy. This refined feature set formed the basis for building predictive models with enhanced reliability and clinical applicability.

### 2.6 Building and Validating Radiomics-based Machine Learning Models

Three machine learning (ML) models were constructed to predict synchronous liver metastases (SLM) in rectal cancer patients using optimal feature subsets, which were chosen through dimensionality reduction. The models were developed using Support Vector Machine (SVM), Random Forest (RF), and Logistic Regression (LR) algorithms. Given their apparent efficacy and widespread application in radiomics and medical imaging research, LR, SVM, and RF have been chosen for predictive modeling. In recognition of its ease of use and interpretability, logistic regression (LR) is an often-used baseline approach for binary classification [29]. Small sample numbers and high-dimensional data, which are prevalent in radiomics, are desirable for Support Vector Machines (SVM); meanwhile, an ensemble learning technique called Random Forest (RF) operates especially well with heterogeneous radiomic data, being that it can capture complicated nonlinear correlations and is resistant to overfitting [30]. Our dataset's linear and nonlinear prediction capabilities may be thoroughly compared according to the application of these three distinct algorithms. To ensure proportional uniformity with the entire set of data, the 137 samples were split into training and test sets in a 7:3 ratio using stratified random sampling. The dataset was split using the train_test_split function from the scikit-learn Python library. The training set was utilized to build the model, while the test set was set aside for independent validation. Three models were built: the Combined Model, incorporating optimal features from both T2WI and DWI sequences; the Combined-Optimal Model, selecting key features from the combined; and the Optimal Model, which identified the most relevant features directly from the primary T2WI and DWI feature sets. The diagnostic performance of the models was assessed by applying several evaluation metrics. The models' overall diagnostic ability was analyzed using the Receiver Operating Characteristic (ROC) curve; classification accuracy was measured by the Area Under the Curve (AUC); the percentage of correctly classified cases was quantified by the Accuracy (ACC); Sensitivity evaluated the model's effectiveness in detecting positive cases, and Specificity determined its ability to identify negative cases. Five-fold cross-validation was conducted on the training set to validate model accuracy, while the test set was used to assess predictive performance based on thresholds established during training. Accuracy and ROC analysis indicated that the model performed the best in terms of diagnosis. Key differences were observed between the Combined-Optimal Model and the Optimal Model. The Combined-Optimal Model selected features from a combined feature set created after feature selection from T2WI and DWI sequences, while the Optimal Model directly selected features from the original T2WI and DWI sequences before feature selection. The accuracy, confusion matrix analysis, and average AUC displayed the clinical importance and dependability of these ML models in predicting SLM for diagnosis and therapy of rectal cancer.

### 2.7 Statistical Analysis

SPSS software (version 22.0, IBM, Chicago, IL, USA) was applied to statistically analyze clinical features. When comparing categorical variables involving gender, stage, location, distance, and circumferential range, the chi-square test was performed. Continuous variables were evaluated using the independent t-test when they followed a normal distribution, whereas the Mann–Whitney U test was applied for variables that did not meet this criterion. Variables having a normal distribution were expressed as mean ± standard deviation (SD). In particular, the Mann-Whitney U test was used to assess tumor size, and the t-test was employed to compare age. Statistical significance was determined by a two-tailed *p*-value of less than 0.05.

Data analysis was performed employing three machine learning (ML) algorithms implemented *via* the Scikit-learn package (https://scikit-learn.org/stable/) in Python. Receiver operating characteristic (ROC) curves serve to assess the prediction capability of the machine learning models. The primary metrics used to evaluate classification performance were the area under the curve (AUC). The Seaborn and Matplotlib libraries in Python (version 3.8) have been used in tandem to create ROC curves. The computation of additional indicators of performance, such as accuracy (Eq. **1**), sensitivity (Eq. **2**), specificity (Eq. **3**), F1-score (Eq. **4**), positive predictive value (PPV), and negative predictive value (NPV), was further made easier by these tools. The classification efficiency of the models was further evaluated using a confusion matrix, offering a visual representation of model performance and allowing the quantification of feature importance.

**Table:** 

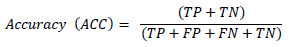	(1)

**Table:** 

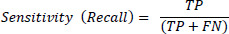	(2)

**Table:** 

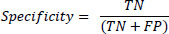	(3)

**Table:** 

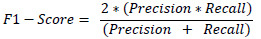	(4)

Where, TP = True Positives; TN = True Negatives; FP = False Positives; FN = False Negatives; and precision is TP / (TP + FP).

## 3 RESULTS

### 3.1 Clinical Features

This study comprised a total of 137 patients diagnosed with rectal cancer, consisting of 93 males and 44 females, with an average (±SD) age of 62.83 ± 11.25 years (range: 33 to 88 years). Among these, 71 patients developed synchronous liver metastases (SLM) within six months, whereas 66 did not. The clinical baseline characteristics of both groups are summarized in Table **2**. The clinical characteristics considered in this study included age, gender, tumor (mrT) staging, lymph node (mrN) staging, tumor size, the distance from the lower edge of the tumor to the anal verge, tumor location, and circumferential range. Among these variables, the mrT stage (*p*-value = 0.012) and the mrN stage (*p*-value = 0.005) were identified as statistically significant, indicating their importance in differentiating between patients with and without SLM. Table **2** illustrates the clinical features and analysis findings.

### 3.2 Selected Features for Model Building

**Fig. (4):**
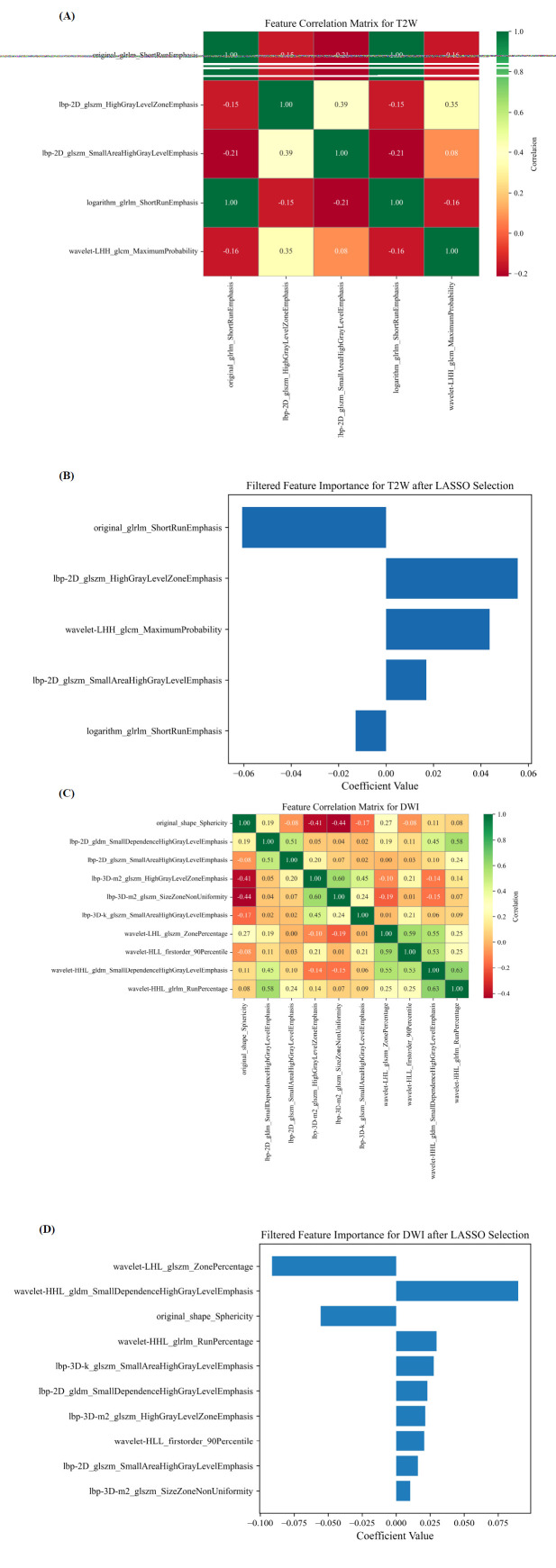
Heatmap showing correlation of the selected features from (**A**) T2WI and (**C**) DWI. Histogram representing coefficients of (**B**) selected T2WI features and (**D**) selected DWI features.

A total of 3,452 radiomic features were extracted, including 1,726 which were obtained from T2WI and 1,726 from DWI. We reduced feature redundancy and identified the most predictive radiomic features from T2W and DWI images by performing Mann-Whitney U test, LASSO-based feature selection, and correlation analysis. The correlation matrix for T2W (Fig. **4A**) showed that majority of selected features exhibit weak correlations (|r| < 0.4), indicating low multicollinearity and suitability for inclusion in predictive modeling. However, original_glrlm_ShortRunEmphasis and logarithm_glrlm_ShortRunEmphasis were highly correlated (r = 1.00), suggesting potential redundancy between these features. Following LASSO regularization (Fig. **4B**), five features were retained with non-zero coefficients. However, logarithm_glrlm_ShortRunEmphasis feature was removed because of high correlation in final selected features (Table **3**). For DWI, the correlation matrix (Fig. **4C**) also showed generally weak to moderate correlations, the value ranging from -0.44 to 0.63. LASSO selection method (Fig. **4D**) identified wavelet-HHL_gldm_SmallDependenceHighGray LevelEmphasis, wavelet-LHL_glszm_ZonePercentage, and original_shape_Sphericity as the most predictive features for DWI. These results underscore the combined significance of texture and shape-related features in capturing meaningful trends from both T2W and DWI modalities. Through this rigorous screening method resulted in 4 features from T2WI and 10 features from DWI were chosen for radiomic signature construction and model development. Figure (**4**) shows their distribution and correlations, and Table **3** lists the optimal features along with their coefficient.

### 3.3 Comparison of Predictive Models Based on T2WI and DWI Radiomic Features

To explore the individual predictive value of each MRI sequence, we constructed separate models using the selected radiomic features from T2WI and DWI images. Table **S1** delivers an overview of their performance. Amongst all machine learning algorithms (LR, SVM, RF), models constructed using DWI features demonstrated a slightly higher degree of predictive ability compared to those constructed using T2WI features. However, the differences were not statistically significant. These findings suggest that both sequences provide complementary but similarly valuable information for predicting SLM, supporting the rationale for developing combined-feature models to potentially improve predictive performance.

### 3.4 Performance Comparison of ML Algorithms in Predicting Synchronous Liver Metastasis: Confusion Matrix Analysis

Table **4** presents the confusion matrices for three predictive model groups (Combined, Combined-Optimal, and Optimal) using three machine learning algorithms (LR, SVM, and RF). The test set comprised 42 patients, including 23 Non-SLM and 19 SLM cases. For the Combined model, the RF achieved the highest performance with 13 true non-SLM predictions and 16 true SLM predictions. The LR and SVM showed similar outcomes, with slightly lower accuracy in predicting both non-SLM and SLM cases. In the Combined-Optimal model, RF again performed well, predicting 13 true non-SLM and 17 true SLM cases, followed by SVM and LR with similar results but lower prediction accuracy. For the Optimal model, RF showed the best performance with 13 true non-SLM and 17 true SLM predictions, while LR and SVM demonstrated comparable outcomes, with the SVM having a slightly higher number of correct SLM predictions. Overall, the RF algorithm consistently outperformed the LR and SVM algorithms across all models.

### 3.5 Predictive Model Development and Diagnostic Efficiency for Synchronous Liver Metastasis

Predictive models for synchronous liver metastasis (SLM) were developed using three machine learning algorithms—logistic regression (LR), support vector machine (SVM), and random forest (RF)—combined with a five-fold cross-validation strategy. Three feature sets were used for model construction: (A) the Combined feature set, consisting of 14 selected features (4 from T2WI and 10 from DWI); (B) the Combined-Optimal feature set, comprising 11 optimal features (4 from T2WI and 7 from DWI) selected from the Combined set; and (C) the Optimal feature set, including 13 features (5 from T2WI and 8 from DWI) chosen from 3452 primary T2WI and DWI features before feature selection. The diagnostic performance of each model was evaluated using metrics such as area under the curve (AUC), accuracy (ACC), sensitivity, specificity, and F1-score, as detailed in Table **5**.

The results revealed that for the Combined model, the RF algorithm achieved the highest AUC of 0.76 (95% CI: 0.61–0.89), with an accuracy of 0.69, sensitivity of 0.84, specificity of 0.61, and F1-score of 0.71 (Table **5**). The LR and SVM algorithms showed similar performance, both with an AUC of 0.70 (95% CI: 0.53–0.85 and 0.52–0.84, respectively), and an accuracy of 0.60. In the Combined-Optimal model, the RF algorithm again outperformed both LR and SVM, achieving an AUC of 0.76 (95% CI: 0.60–0.89), with an accuracy of 0.71, sensitivity of 0.89, specificity of 0.57, and F1-score of 0.74 (Table **5**). The LR and SVM models recorded AUCs of 0.70 and 0.69, respectively, with lower overall performance metrics.

**Fig. (5):**
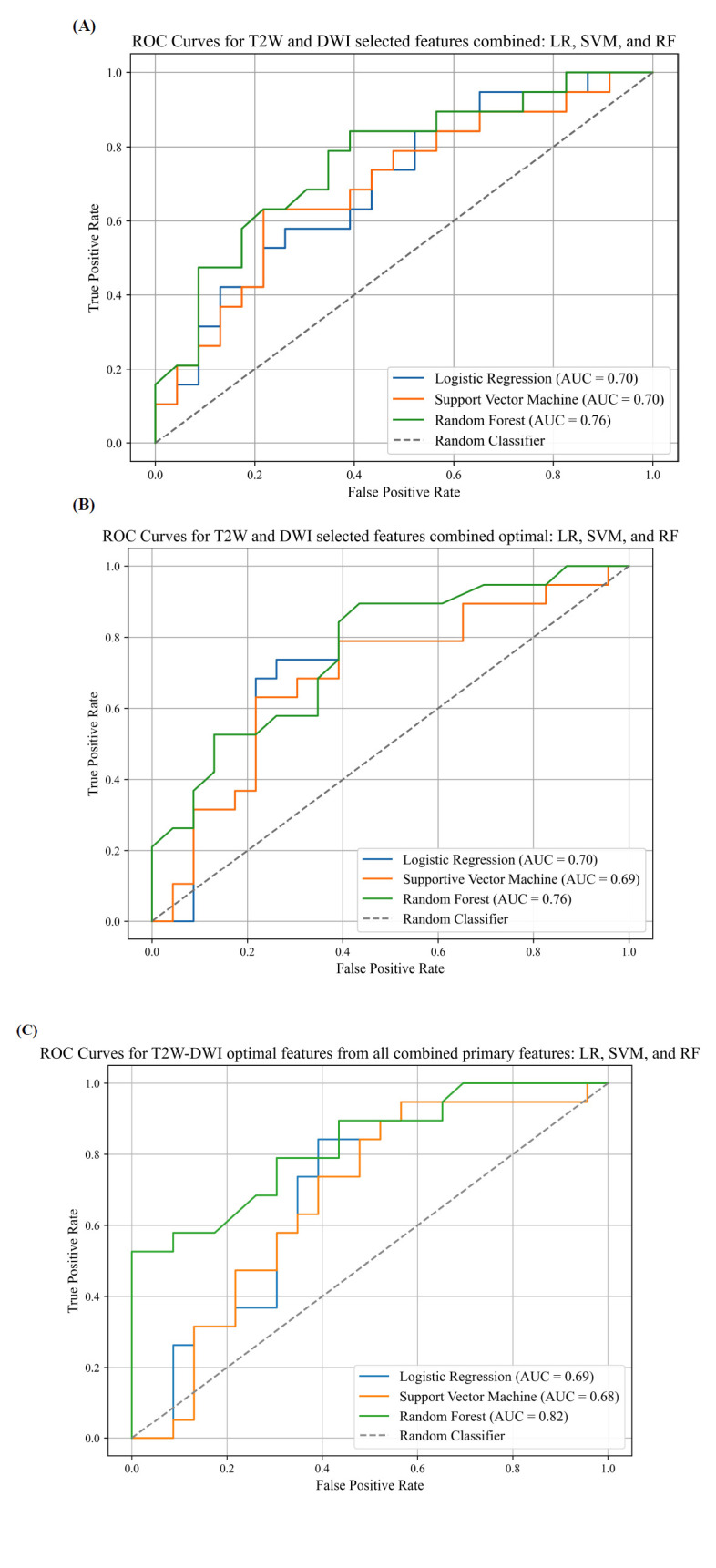
Comparison of ROC curves for the LR, SVM, and RF algorithms across three groups of models. A five-fold cross-validation approach and three machine learning algorithms were employed to construct predictive models for synchronous liver metastasis (SLM) using different feature sets: (**A**) the Combined feature set (selected T2WI and DWI features), (**B**) the Combined-Optimal feature set (optimal features selected from the combined feature set), and (**C**) the Optimal feature set (optimal features selected from primary T2WI and DWI combined before feature selection). In each ROC curve, the blue, orange, and green lines represent LR, SVM, and RF algorithms, respectively.

The Optimal model demonstrated the best diagnostic performance across all groups. The RF algorithm yielded the highest AUC of 0.82 (95% CI: 0.66–0.93), with an accuracy of 0.71, sensitivity of 0.53, specificity of 1.00, and F1-score of 0.74 (Table **5**). The SVM and LR algorithms had moderate performance, with AUCs of 0.68 and 0.69, respectively. These results confirm that the RF algorithm consistently outperformed the LR and SVM algorithms across all models, highlighting its superior predictive capability for SLM and underscoring its potential value in clinical decision-making. Figure **5** illustrates the ROC analysis for the three models.

The variations in AUC values between the three classifiers can be observed in the ROC curves. The AUC values for LR and SVM are comparable in Fig. (**5A** and **5B**), even though RF succeeds in a somewhat higher AUC of 0.76. RF performs most ideally with an AUC of 0.82 in Fig. (**5C**), which relies on the best possible combination of each of the primary features, followed by LR (0.69) and SVM (0.68). This demonstrates that RF performs higher than the other algorithms regularly, particularly where its comprehensive feature set is employed.

## 4 DISCUSSION

The ultimate objective of the clinical practice of detecting and managing rectal cancer is to identify liver metastases from cancer at an early stage, particularly for forecasting SLM. In this study, predictive models for synchronous liver metastasis (SLM) were built using three machine learning algorithms: LR, SVM, and RF. A five-fold cross-validation approach was employed to ensure robust model evaluation. Across all models, the RF algorithm revealed superior predictive performance compared to LR and SVM. LR and SVM recorded less valuable AUCs of 0.69 and 0.68 for the optimal model; however, RF achieved the greatest AUC of 0.82 with strong diagnostic measures. Confusion matrices demonstrate that RF accurately predicted SLM cases with offering a balanced sensitivity and specificity. Meanwhile, LR and SVM exhibited moderate performance and consistently fell short of RF's accuracy. In addition, the advantages of various algorithms vary. A study that evaluated the predictive power of logistic regression along with certain machine learning methods [31] revealed that logistic regression's predictive efficiency was quite impressive. We discovered that the RF algorithm was the most effective in the findings we obtained. We examined how well it predicted outcomes when compared with alternative algorithms. A previous study reported AUCs of 0.73 and 0.75 for an MRI-radiomics model in the training and validation sets, with a radiomics-clinical nomogram achieving an AUC of 0.809 [32], highlighting the utility of MRI-radiomics in predicting the chemotherapeutic response in SLM patients. In comparison, our study demonstrated a higher AUC of 0.82 using the RF algorithm, which also showed superior diagnostic performance with an accuracy of 0.71. Moreover, a recent multimodal radiomics study incorporating MRI, CT, and clinical features achieved a maximum AUC of 0.82 for MLM prediction, while the MRI-only and clinical models yielded lower AUCs of 0.68 and 0.62, respectively [33]. In contrast, our MRI-based RF model attained an AUC of 0.82 without relying on CT or clinical data, highlighting the efficiency of our radiomic feature selection and machine learning strategy for accurate SLM prediction.

We propose that relatively small datasets with dichotomous variables, such as those in our study, can be computed with the RF, LR, and SVM algorithms. Model stability and prediction performance may be improved by implementing ideal machine learning algorithms. Prior research has demonstrated the effectiveness of SVM and LR in constructing reliable models for various tumors, with SVM reportedly outperforming LR [34, 35]. However, in our study, we observed that the Random Forest (RF) algorithm outperformed both LR and SVM in the Model-Optimal group, while their performances were comparable across the other two model groups. Moreover, the results we obtained suggest that the RF algorithm is far superior for the MRI-enhanced sequence. From the perspective of the algorithms employed, the models demonstrated stability. Although only three algorithms were evaluated in this study, future research ought to explore additional algorithms for a more comprehensive assessment. Five-fold cross-validation has been implemented for this research to evaluate our radiomics-based machine learning models' prediction capabilities. As suggested by earlier research [36], this method minimizes the risk of overfitting while optimizing model performance, which is what makes radiomics research has adopted it extensively. Earlier studies [37, 38] proportionately split patients into training and validation sets at random, which unavoidably generated selection bias; five-fold cross-validation is particularly appropriate for small-sample model building than prior approaches.

The significant role of the radiomics signature (RS) as a vital aid for forecasting the possibility of SLM in patients with primary rectal cancer is highlighted by this study. A metric that is exclusively dependent on imaging, which is made up of radiomic features, is called a radiomics signature (RS). Developing a radiomics model based solely on radiomic features, without incorporating clinical data, provides several benefits. First, it allows for early prediction of therapeutic response or disease progression based entirely on imaging features, enabling more timely clinical decision-making steps compared to relying purely on clinical data. Second, excluding clinical data simplifies the model, making it easier and quicker to create, implement, and interpret. This simplified approach is particularly advantageous in situations where clinical data is inconsistent, unavailable, or challenging to integrate. Lastly, a radiomics-only model highlights the performance of imaging biomarkers, which is especially valuable for studies aimed at enhancing imaging technologies or advancing imaging-based diagnostic methods. This finding aligns with prior research [24, 39], which demonstrated that radiomics signatures (RS) obtained from CT or MRI can effectively stratify high-risk cancer patients. For instance, Huang *et al*. [39] developed a radiomics signature model based on multiparametric MRI (mp-MRI), incorporating T2WI, DWI, and CE-T1WI, to predict outcomes in locally advanced rectal cancer. The model achieved AUCs of 0.83, 0.81, and 0.82 in the training, internal validation, and external validation cohorts, respectively. Moreover, the RS showed clinical utility in predicting 3-year disease-free survival (DFS) in patients with locally advanced rectal cancer [39]. Previous studies [40] on rectal cancer have also recommended that the MRI radiomics signature could serve as an efficient biomarker for predicting long-term positive outcomes. Although these studies did not specifically explore SLM stratification within the context of rectal cancer radiomics investigations.

Within six months, all 71 individuals in this research acquired SLM. There are presently a few research studies that use MRI radiomics to predict SLM from rectal cancer. Nevertheless, to predict metachronous liver metastasis (MLM), Li *et al*. [41] developed a radiomics model employing contrast-enhanced CT of colorectal cancer. They reported AUC values of 0.79 ± 0.08 for the internal validation cohort and 0.72 ± 0.07 for the external validation cohort. One meta-analysis revealed that colorectal liver metastases (LM) achieved detection sensitivity of 63%-80% in contrast-enhanced CT, 76%-85.7% in routine MRI, and 51%-90% in FDG PET-CT [42]. Thus, the likelihood of early SLM detection in rectal cancer patients would increase with screening for high-risk imaging indicators.

In our study, we noticed that machine-learning models based on radiomic features extracted from MRI provided enhanced predictive performance compared to traditional assessments. This aligns with previous research demonstrating that statistical models incorporating tumor characteristics can outperform human experts in prognosis and prediction [43]. Regardless of the potential of radiomics in clinical decision support, inherent obstacles remain. Variances in radiomic features due to differences in imaging scanners [44], tumor segmentation methods [45], reconstruction algorithms, and feature discretization have been widely reported. Addressing these sources of variability is essential for ensuring the robustness of radiomics-based analyses. In this regard, feature normalization or standardization can help mitigate batch effects and improve model generalizability. In this study, we developed and validated a radiomics model for predicting SLM in rectal cancer patients using high-resolution T2WI and DWI, which are standard modalities for rectal cancer assessment. Restricted diffusion of water molecules in the tumor cell microenvironment can be identified by high-b value DWI, which may provide further insight into the internal heterogeneity of the tumour [46]. The proposed model, based on radiomic features extracted from preoperative MRI scans, serves as a practical tool for identifying patients who might benefit from further liver imaging. Three variations of the model were evaluated: the Combined model (features selected from T2WI and DWI), the Combined-Optimal model (optimal features selected from the combined feature set), and the Optimal model (optimal features selected from primary T2WI and DWI combined before feature selection). Among these, the optimal model outperformed the others in terms of prediction. This underscores how important it is to choose the most relevant features from both T2WI and DWI with the goal of boosting accuracy. Although combining radiomic features from T2WI and DWI was expected to improve performance, our results showed only modest gains. The Combined-Optimal model using Random Forest achieved an AUC of 0.76, which was lower than the Optimal model’s AUC of 0.82. This implies that careful feature selection is now more essential for enhancing predictive accuracy than simply merging features from multiple MRI sequences. To predict lymphatic vascular infiltration in rectal cancer, Zhang *et al*. [47] established a rad-score model applying enhanced CT as well as multi-sequence MRI (T2WI + DWI). The findings indicated that the mp-MRI model outperformed other single-sequence models in terms of performance. Consequently, a multimodality radiomics predictive model could assist in the early identification and subsequent evaluation of patients with rectal cancer [48].

## 5 LIMITATIONS AND FUTURE DIRECTIONS

There are various limitations in this study. First of all, the study is a single-center retrospective research with a limited number of participants, with the lack of external validation utilizing independent multi-center datasets is one of its main limitations. This limits our capacity to thoroughly evaluate the model’s generalizability and robustness across diverse patient populations and varying imaging scenarios. To mitigate this, we intend to collaborate with additional centers in future research to obtain larger and more diverse datasets, enabling comprehensive external validation and potential refinement of the model. These steps are vital for advancing the medical use and wider implementation of radiomics-based predictive models. Secondly, this study did not employ a layer-by-layer delineation of regions of interest (ROIs) across all tumor display levels. Despite being thorough, the layer-by-layer delineation method is laborious and not always feasible in a hectic clinical setting, even if it delivers more information regarding tumor heterogeneity. The future of image segmentation relies on automatic segmentation algorithms, which minimize the need for human judgment. Thirdly, treatment plans that could influence the incidence of synchronous liver metastases (SLM) in patients having rectal cancer were not taken into account because of the lack of comprehensive follow-up data. Moreover, the short follow-up period might have prevented the detection of liver metastases that appeared over six months after the initial rectal cancer diagnosis. We recognize that even though the 6-month cutoff for recognizing SLM is commonly applied, there is variability in the literature, with definitions ranging from 3 to 12 months, potentially affecting reported incidence rates and prognostic assessments [3]. Standardizing this classification in future research may help improve uniformity and comparability across studies. Fourthly, the study relied solely on radiomics features extracted from T2WI and DWI for model construction. Due to sample size limits and insufficient data, other sequences, like apparent diffusion coefficient (ADC) maps, and clinical characteristics, such as carcinoembryonic antigen (CEA), were excluded. Future research integrating ADC maps and CEA has the potential to enhance the predictive accuracy and overall utility of radiomics models. The outcomes show that combining features from various sequences may not always result in superior accuracy and can cause overfitting or redundancy if not handled appropriately. The findings underline the necessity of skilled feature fusion approaches, including dimensionality reduction, hierarchical selection, or deep learning-based fusion methods, in order to more appropriately access relevant data. This profound insight adds to the depth of knowledge already in existence and guides future investigations meant to maximize multi-sequence radiomic modeling. This study used LR, SVM, and RF algorithms because of their well-established application and interpretability in radiomics research. Nevertheless, we comprehend the limitation in the diversity of models applied. To potentially boost predicted accuracy, future research ought to explore a broader variety of ML algorithms, such as KNN, Decision Trees, and Naive Bayes. In addition, more advanced ensemble methods beyond RF, as well as deep learning techniques, could better reflect complex nonlinear relationships within radiomic features. While deep learning has shown excellent performance in many medical imaging applications, it generally demands big datasets to minimize overfitting and to achieve strong generalization. Due to the relatively small sample size in our study (n = 137), we decided not to apply deep learning methods to avoid the risk of overfitting and ensure more stable predictive results. We openly acknowledge this limitation to explain our methodological choices and emphasize the importance of larger datasets in future studies to fully leverage deep learning techniques. Gaussian filtering was applied to minimize noise and improve consistency across scans. Although it may influence the distribution of radiomic features, particularly texture-related metrics, future studies will evaluate its effect on feature reliability and model accuracy.

## CONCLUSION

The potential benefit of leveraging radiomic features from T2WI and DWI MRI sequences for developing machine learning-based predictive models for early identification of synchronous liver metastases (SLM) in patients with rectal cancer is proven in this study. The optimal feature set-based predictive model demonstrated the highest accuracy for SLM detection, with the RF algorithm outperforming LR and SVM by consistently achieving the best AUC and balanced diagnostic performance. The incorporation of radiomics and RF-based models conveys a promising, non-invasive approach for enhancing early detection and risk stratification of SLM, which could help with more reliable clinical decision-making and individualized treatment planning for patients with rectal cancer. The present research additionally highlights how crucial the method of feature selection is for strengthening model performance and minimizing dimensionality. Furthermore, while these results offer credibility to the therapeutic usefulness of radiomics-driven machine learning models, additional studies using larger multi-center datasets and external validation would be helpful to improve model generalizability, lessen the variability, accessibility, and clinical implementation.

## Figures and Tables

**Fig. (1):**
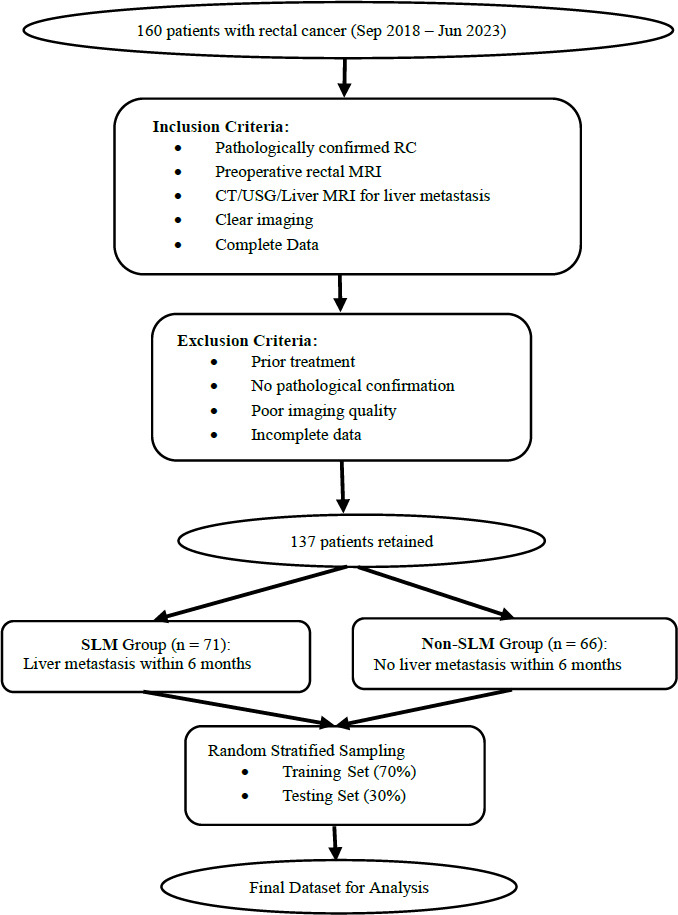
Schematic representation of patient enrollment.

**Fig. (2):**
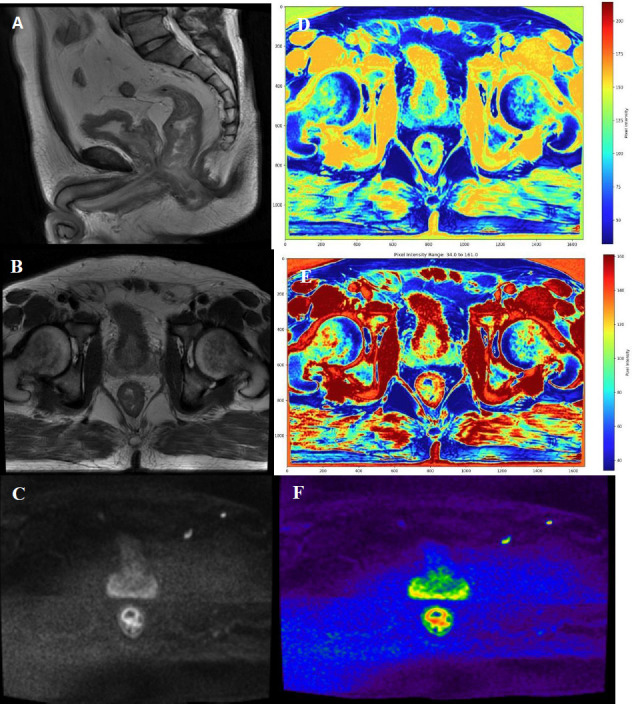
Colormap representation of MRI in a 56-year-old male with T2N1 stage rectal cancer: (**A**) Sagittal T2WI showing a tumor in the low rectum; (**B**) Oblique axial T2WI highlighting the rectal tumor; (**C**) Oblique axial DWI of the same tumor; (**D**) Default colormap representation of the tumor on an axial T2WI; (**E**) Alternative colormap representation of the tumor on axial T2WI; (**F**) Rainbow colormap representation of the tumor on axial DWI. This image serves as a visual representation; however, radiomic feature extraction was conducted using the original grayscale MRI data.

**Fig. (3):**
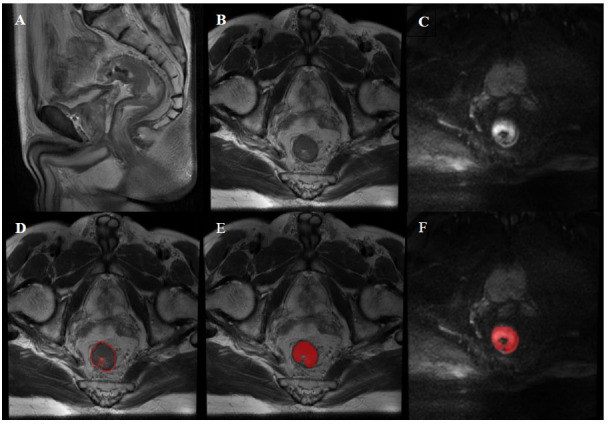
A schematic illustration of the ROI placement method in a 63-year-old male with T3N1 stage rectal cancer: Image (**A**) Sagittal T2WI showing a tumor located in the mid and upper rectum; Image (**B**) Oblique axial T2WI image of the rectal tumor; Image (**C**) Oblique axial DWI of the rectal tumor for the same patient; Image (**D**) Delineation of the region of interest (ROI) using red lines on an axial T2WI image. Image (**E**) Manual drawing of a 2D ROI on the largest cross-sectional area of the tumor in axial T2WI, covering the entire lesion on a single slice. Image (**F**) Manual ROI placement on the largest cross-sectional area of the tumor in DWI, ensuring complete coverage of the lesion on one slice.

**Table 1:** MRI sequence and parameter overview.

**Equipment**	**Parameters (MRI)**	**T2WI**			**DWI**
		**Axial Oblique**	**Coronal Oblique**	**Sagittal**	**Axial**
-	TR, ms	5990	5373	5373	4881
-	TE, ms	125.7	125.7	125.7	72
-	FOV, mm	200	200	200	280
-	Slice thickness, mm	5	5	5	5
GE Discovery MR750 3.0T	Slice spacing, mm	6	6	6	6
-	Matrix	320 x 320	320 x 320	320 x 320	280 x 280
-	Acquisition time, min	2.5	2.5	2	2
-	Bandwidth, Hz/pixel	325	325	488	1953
-	b-values	-	-	-	1000

**Table 2:** Evaluation of clinical information between the SLM and non-SLM groups through statistical analysis.

**Clinical Characteristics**	**SLM Group (n=71)**	**Non-SLM Group (n=66)**	***p*-value**
**Age (mean ± SD)**	63.72±11.33	61.89±11.18	0.35
**Gender (%)**	-	-	0.51
Male	50 (70.42%)	43 (65.15%)	-
Female	21 (29.58%)	23 (34.85%)	-
**mrT stage (%)**	-	-	0.01
T2	4 (5.63%)	15 (22.73%)	-
T3	53 (74.65%)	43 (65.15%)	-
T4	14 (19.72%)	8 (12.12%)	-
**mrN stage**	-	-	0.01
N0	4 (5.63%)	12 (18.18%)	-
N1	23 (32.39%)	30 (45.45%)	-
N2	44 (61.97%)	24 (36.36%)	-
**Tumor diameter,** mean ± SD, cm	7.01±2.88	7.04±2.91	0.11
**Tumor location** (%)	-	-	0.29
Upper rectum	13 (18.31%)	13 (19.70%)	-
Middle rectum	37 (52.11%)	26 (39.39%)	-
Lower rectum	21 (29.58%)	27 (40.91%)	-
**Distance (cm)**	-	-	0.09
<5	12 (16.90%)	21 (31.82%)	-
5-10	49 (69.01%)	40 (60.61%)	-
>10	10 (14.08%)	5 (7.57%)	-
**Circumferential range** (n)	-	-	0.16
1/4-1/2	17 (23.94%)	23 (34.85%)	-
1/2-3/4	31 (43.66%)	19 (28.79%)	-
3/4-1	23 (32.39%)	24 (36.36%)	-

**Abbreviation: **SD = standard deviation.

**Table 3:** Relevant features selected from T2WI, DWI, combined-optimal radiomic features, and primary optimal features.

**Features Group**	**Key Features**	**Coefficient Value**
T2WI selected features set (n=4)	original_glrlm_ShortRunEmphasis	-0.060676
-	lbp-2D_glszm_HighGrayLevelZoneEmphasis	0.055537
-	wavelet-LHH_glcm_MaximumProbability	0.043665
-	lbp-2D_glszm_SmallAreaHighGrayLevelEmphasis	0.016997
DWI selected features set (n=10)	wavelet-LHL_glszm_ZonePercentage	-0.091389
-	wavelet-HHL_gldm_SmallDependenceHighGrayLevelEmphasis	0.089822
-	original_shape_Sphericity	-0.055560
-	wavelet-HHL_glrlm_RunPercentage	0.029717
-	lbp-3D-k_glszm_SmallAreaHighGrayLevelEmphasis	0.027646
-	lbp-2D_gldm_SmallDependenceHighGrayLevelEmphasis	0.022900
-	lbp-3D-m2_glszm_HighGrayLevelZoneEmphasis	0.021389
-	wavelet-HLL_firstorder_90Percentile	0.020684
-	lbp-2D_glszm_SmallAreaHighGrayLevelEmphasis	0.015947
-	lbp-3D-m2_glszm_SizeZoneNonUniformity	0.010360
Selected Combined Optimal Features set (n=11)	original_glrlm_ShortRunEmphasis_T2W	-0.060676
-	lbp-2D_glszm_HighGrayLevelZoneEmphasis_T2W	0.055537
-	lbp-2D_glszm_SmallAreaHighGrayLevelEmphasis_T2W	0.016997
-	wavelet-LHH_glcm_MaximumProbability_T2W	0.043665
-	original_shape_Sphericity_DWI	-0.05556
-	lbp-3D-m2_glszm_HighGrayLevelZoneEmphasis_DWI	0.021389
-	lbp-3D-k_glszm_SmallAreaHighGrayLevelEmphasis_DWI	0.027646
-	wavelet-LHL_glszm_ZonePercentage_DWI	-0.09138
-	wavelet-HLL_firstorder_90Percentile_DWI	0.020684
-	wavelet-HHL_gldm_SmallDependenceHighGrayLevelEmphasis_DWI	0.089822
-	wavelet-HHL_glrlm_RunPercentage_DWI	0.029717
Selected Optimal features from the primary T2WI and DWI features set (n=13)	wavelet-LHL_glszm_ZonePercentage_DWI	-0.062258
-	wavelet-HHL_gldm_SmallDependenceHighGrayLevelEmphasis_DWI	0.056023
-	original_shape_Sphericity_DWI	-0.048363
-	wavelet-LHH_glcm_MaximumProbability_T2W	0.047204
-	original_glrlm_ShortRunEmphasis_T2W	-0.037768
-	lbp-2D_glszm_HighGrayLevelZoneEmphasis_T2W	0.035954
-	wavelet-HLL_firstorder_90Percentile_DWI	0.030305
-	lbp-3D-k_glszm_SmallAreaHighGrayLevelEmphasis_DWI	0.023594
-	original_glrlm_RunLengthNonUniformity_DWI	0.023320
-	wavelet-HHL_gldm_LargeDependenceEmphasis_DWI	-0.020106
-	lbp-2D_glszm_SmallAreaHighGrayLevelEmphasis_T2W	0.019108
-	lbp-3D-m2_glszm_HighGrayLevelZoneEmphasis_DWI	0.015868
-	lbp-2D_ngtdm_Busyness_T2W	0.01204

**Note: **Each feature name is presented in the format: filter_feature group_feature. - Features in the “Combined-Optimal” feature set were derived from the selected radiomic features of T2WI and DWI combined. - Features in the “Optimal” feature set were selected from the entire pool of 3452 radiomic features (primary T2WI and DWI combined before feature selection).

**Table 4:** Performance evaluation with confusion matrix results for three model groups.

**Predictive Model**	-	**Predicted_NonSLM**	**Predicted_SLM**
Model Combined-LR	True_NonSLM	9	14
-	True_SLM	3	16
Model Combined-SVM	True_NonSLM	8	15
-	True_SLM	2	17
Model Combined-RF	True_NonSLM	13	10
-	True_SLM	3	16
Model Combined Optimal-LR	True_NonSLM	8	15
-	True_SLM	3	16
Model Combined Optimal-SVM	True_NonSLM	8	15
-	True_SLM	2	17
Model Combined Optimal-RF	True_NonSLM	13	10
-	True_SLM	2	17
Model Optimal-LR	True_NonSLM	11	12
-	True_SLM	3	16
Model Optimal-SVM	True_NonSLM	12	11
-	True_SLM	3	16
Model Optimal-RF	True_NonSLM	13	10
-	True_SLM	2	17

**Note: **LR stands for logistic regression; SLM represents synchronous liver metastasis. Model Combined is the model based on the combined relevant feature set derived from selected T2WI and DWI features. Model Combined-Optimal is the model developed using the optimal feature subset extracted from the combined relevant feature set of T2WI and DWI. Model Optimal is the model constructed using the optimal feature subset from the primary T2WI and DWI features. SVM is a support vector machine, and RF is a random forest.

**Table 5:** Evaluation of the diagnostic effectiveness of predictive models across three algorithms.

**Predictive Models**	**ML** **Algorithms**	**AUC** **(Test)**	**(95% CI)**	**ACC**	**Sensitivity**	**Specificity**	**F1-score**
Model Combined	LR	0.70	(0.53, 0.85)	0.60	0.84	0.48	0.65
	SVM	0.70	(0.52, 0.84)	0.60	0.63	0.78	0.67
	RF	0.76	(0.61, 0.89)	0.69	0.84	0.61	0.71
Model Combined-Optimal	LR	0.70	(0.53, 0.86)	0.57	0.74	0.74	0.64
	SVM	0.69	(0.52, 0.85)	0.60	0.63	0.78	0.67
	RF	0.76	(0.60, 0.89)	0.71	0.89	0.57	0.74
Model Optimal	LR	0.69	(0.52, 0.85)	0.64	0.84	0.61	0.68
	SVM	0.68	(0.51, 0.84)	0.67	0.95	0.43	0.70
	RF	0.82	(0.66, 0.93)	0.71	0.53	1.00	0.74

**Note: **The values for AUC (Area Under the Curve), ACC (Accuracy), and CI (Confidence Interval) are provided, with the CI range indicated in parentheses. The comparison includes three algorithms: Logistic Regression (LR), Support Vector Machine (SVM), and Random Forest (RF). Model Combined represents the predictive model using the combined relevant feature set from T2WI and DWI features. Model Combined-Optimal is based on the optimal feature set derived from the combined relevant features, while Model Optimal is developed using the optimal feature set from the combined primary T2WI and DWI features prior to feature selection.

## Data Availability

All data generated or analyzed during this study are included in this published article.

## LIST OF ABBREVIATIONS

ADC: Apparent diffusion coefficient
AI: Artificial intelligence
AUC: Area under the curve
CI: Confidence interval
CRC: Colorectal cancer
CT: Computed tomography
DCE MRI: Dynamic contrast-enhanced magnetic resonance imaging
DWI: Diffusion-weighted imaging
GLCM features: Grey level co-occurrence matrix features
GLDM features: Grey level dependence matrix features
GLRLM features: Grey level run length matrix features
GLSZM features: Grey level size zone matrix features
MRI: Magnetic resonance imaging
NGTDM features: Neighboring grey tone difference matrix features
PACS: Picture archive and communication system
PET: Positron emission tomography
T2WI: T2-weighted imaging

## STANDARDS OF REPORTING

STROBE guidelines were followed
