# Supplementary material for: Comparison of Radiomic Features from Different MRI Sequences for Predicting Synchronous Liver Metastases after Rectal Cancer
Source: Curr Med Imaging. 2025 Nov 28;22:e15734056399652. doi: 10.2174/0115734056399652251029204023 (PMC13312386; doi:10.2174/0115734056399652251029204023)
Supplement: Supplementary file 1 [file CMIM-22-E15734056399652_SD1.pdf]

1875-6603/26 Send Orders for Reprints to reprints@benthamscience.net

BENTHAM SCIENCE

CrossMark

Current Medical Imaging

Content list available at: https://benthamscience.com/journals/cmir

Supplementary Material

Comparison of Radiomic Features from Different MRI Sequences for Predicting Synchronous Liver Metastases after Rectal Cancer

Apekshya Singh[1], Sheng-Ming Shi[1], Han Liu[1], Yu-peng Wu[1], Yuhang Wang[1], Jiayi Xie[1] and Xiao-Fu Li[1,*]

[1]*Department of Magnetic Resonance Imaging, The Second Affiliated Hospital, Harbin Medical University, Harbin 150086, Heilongjiang Province, China*

Combined T2W-DWI selected features:

A total of 15 features were initially selected; however, 14 features were retained for the combined model building after removing highly correlated variables (correlation > ~0.9). Specifically, the feature 'logarithm_glrlm_ShortRun Emphasis_T2W' was excluded to reduce redundancy, resulting in a final dataset with 14 features.

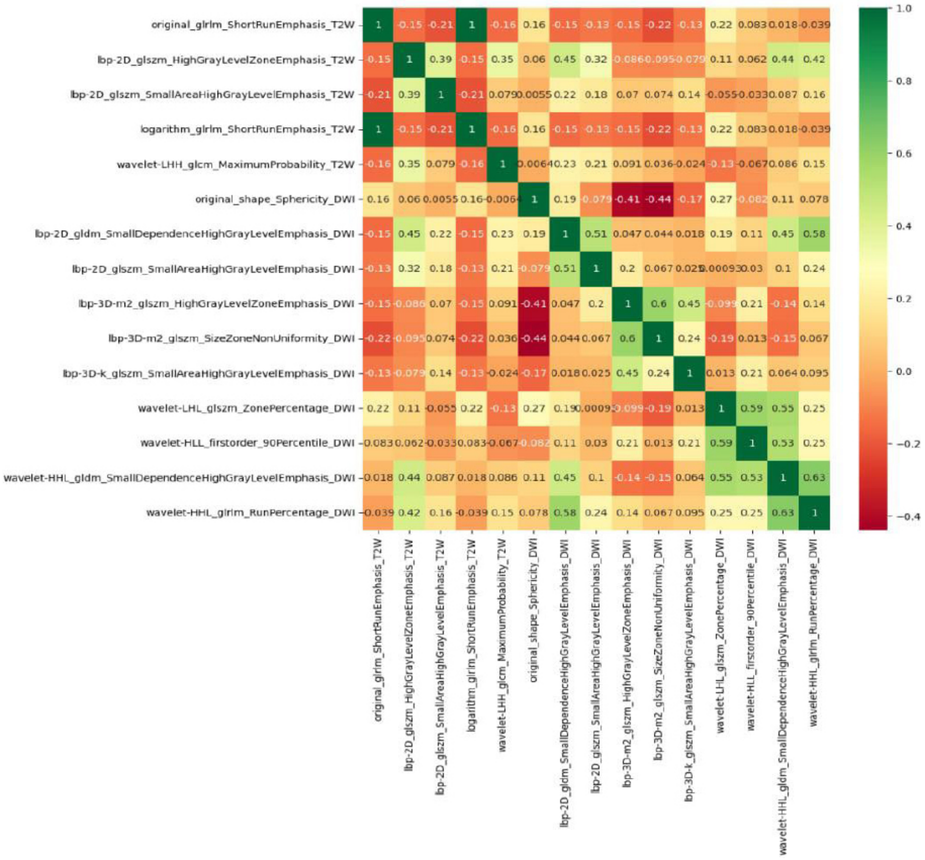

Fig. (S1). Correlation plot of selected features from T2WI and DWI.

DOI: 10.2174/0115734056399652251029204023, 2026, *22*, e15734056399652

Optimal features from combined T2W-DWI selected features:

The LASSO method was applied to select the most important features from the combined dataset. The dataset was then split into training and testing sets using a 70:30 ratio. The training set comprised 95 samples with 14 features, while the test set included 42 samples with the same 14 features. The corresponding target variable had 95 samples in the training set and 42 samples in the test set.

Selected Combined Optimal Features by LASSO:

'original_glrlm_ShortRunEmphasis_T2W', 'lbp-2D_glszm_HighGrayLevelZoneEmphasis_T2W', 'lbp-2D_glszm_SmallAreaHighGrayLevelEmphasis_T2W', 'wavelet-LHH_glcm_MaximumProbability_T2W',

'original_shape_Sphericity_DWI', 'lbp-3D-m2_glszm_HighGray LevelZoneEmphasis_DWI', 'lbp-3D-k_glszm_SmallAreaHighGrayLevelEmphasis_DWI', 'wavelet-LHL_glszm_ZonePercentage_DWI', 'wavelet-HLL_firstorder_90 Percentile_DWI', 'wavelet-HHL_gldm_SmallDependence HighGrayLevelEmphasis_DWI', 'wavelet-HHL_glrlm_Run Percentage_DWI'

Optimal features selected from the primary T2WI and DWI features

Highly correlated variables (correlation > ~0.9) were removed to reduce redundancy and improve model performance. Specifically, 'logarithm_glrlm_ShortRun Emphasis_T2W' and 'wavelet-HHL_gldm_LargeDependence HighGrayLevelEmphasis_DWI' were excluded, resulting in a final dataset containing 13 selected features for building the optimal model.

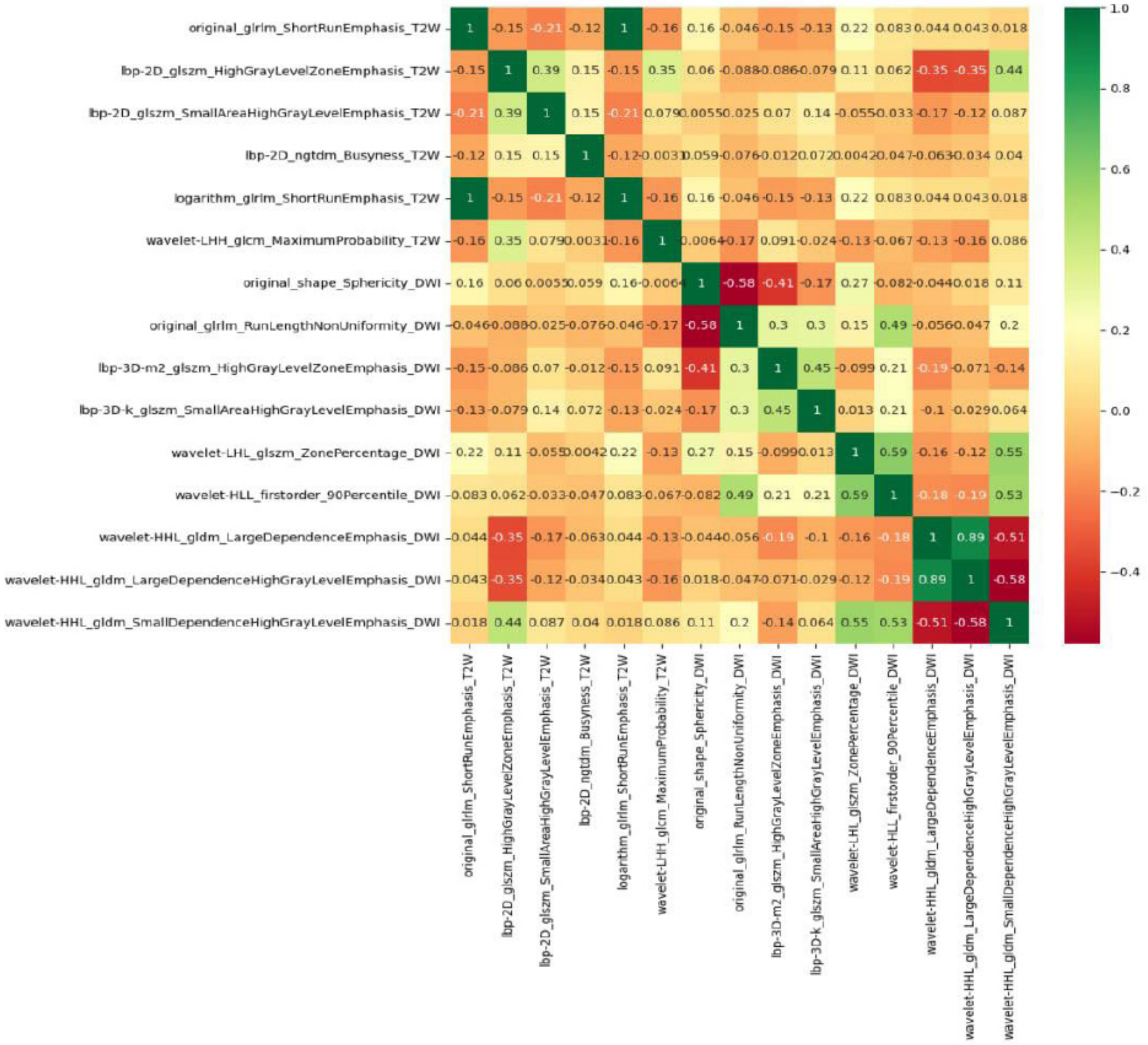

Fig. (S2). Correlation plot of selected features from primary T2WI and DWI features.

LASSO Coefficient Path Plot:

LASSO coefficient paths for radiomic features extracted from DWI sequences. Each curve in Figure S3 represents the trajectory of a feature's coefficient as a function of the regularization parameter alpha. As alpha increases, coefficients of less informative features shrink toward zero. Features with non-zero coefficients at the selected alpha (~0.03), determined by five-fold cross-validation, were retained for predictive modeling.

LASSO Cross-Validation MSE Plot:

To reduce dimensionality and retain the most predictive features, we applied LASSO (Least Absolute Shrinkage and Selection Operator) regression after standardizing all input variables. A sequence of alpha values ranging from 0.01 to 10 was assessed *via* five-fold cross-validation, optimizing model performance based on the cross-validated negative mean squared error (MSE). As shown in the cross-validation plot Figure S4, the optimal alpha was approximately 0.03. This alpha was subsequently used to fit the final LASSO model, retaining features with non-zero coefficients. To further refine the selection and remove variables with minimal predictive value, features with coefficients between –0.01 and +0.01 were excluded.

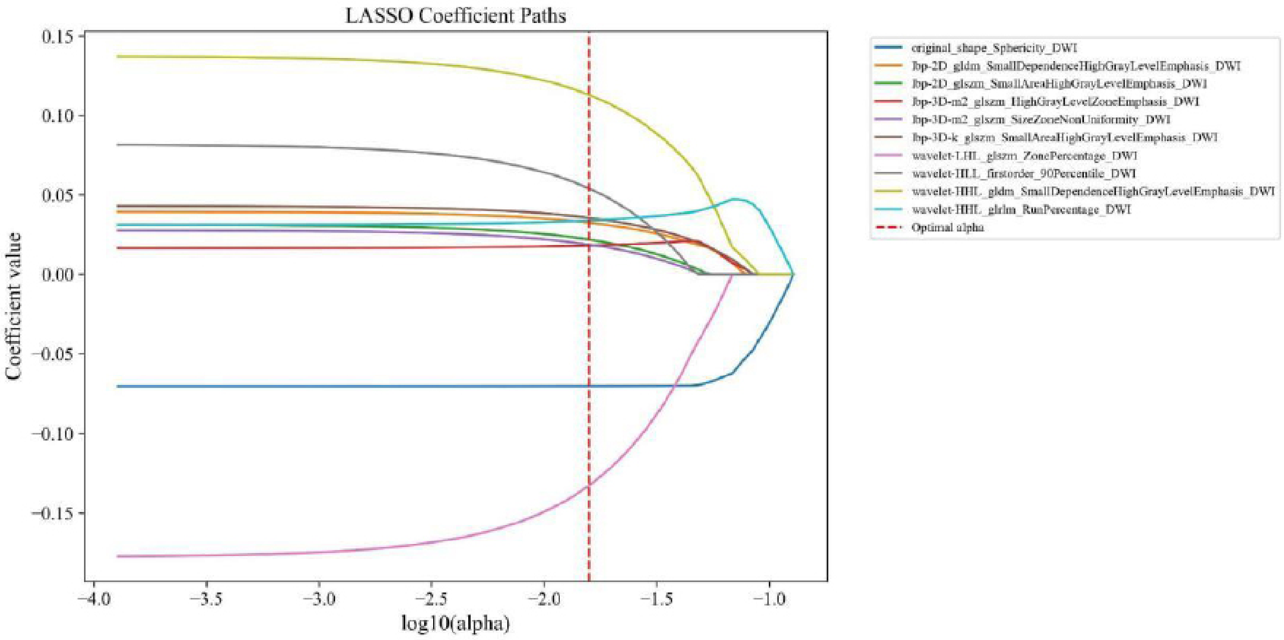

Fig. (S3). LASSO coefficient path plot for DWI radiomic features.

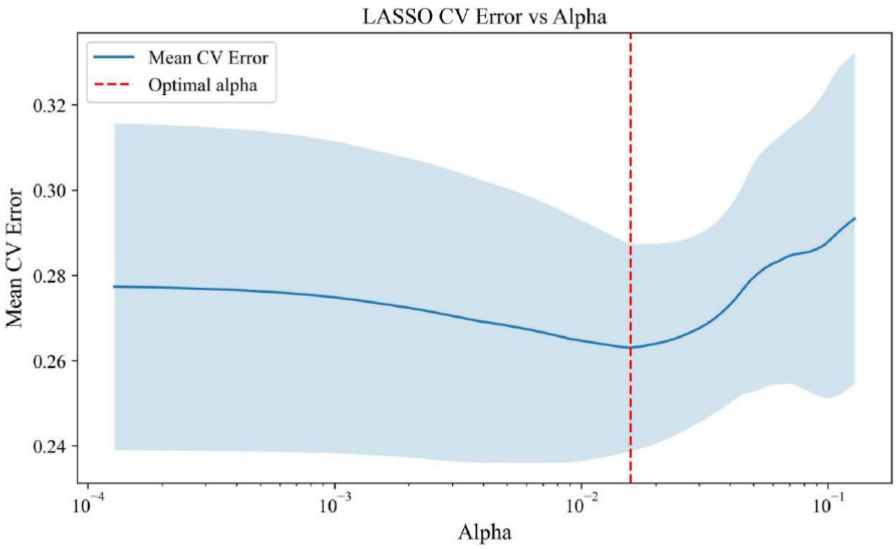

Fig. (S4). Cross-validated MSE curve for LASSO regression on DWI-derived radiomic features..

Comparison of Predictive Models Based on T2WI and DWI Radiomic Features

In addition to showcasing the radiomic features selected from T2WI and DWI sequences (Table 3, Figure 4), we conducted a comparative evaluation of the predictive performance of models developed separately using selected features from each sequence. The outcomes of this analysis are presented in Table S1 (Supplementary Material). The Random Forest model based on DWI features achieved an AUC of 0.70 (95% CI: 0.54–0.86), whereas the model using T2WI features attained an AUC of 0.58 (95% CI: 0.39–0.77). AUCs for SVM and LR models that used T2WI features were 0.63, whereas those of their DWI equivalents were 0.65 and 0.61, respectively. While DWI-based models demonstrated slightly better performance overall, the differences between the two sequences were not statistically significant. These findings suggest that both sequences provide complementary but similarly valuable information for predicting synchronous liver metastases (SLM), supporting the rationale for developing combined-feature models to potentially improve predictive performance.

Table S1. Performance comparison of machine learning models constructed using selected radiomic features from DWI and T2WI sequences.

Sequence	Algorithm	AUC	95% CI	Sensitivity	Specificity
**T2WI**	LR	0.63	(0.45, 0.79)	0.95	0.35
**T2WI**	SVM	0.63	(0.46, 0.80)	0.79	0.48
**T2WI**	RF	0.58	(0.39, 0.77)	0.32	0.91
**DWI**	LR	0.65	(0.46, 0.82)	0.74	0.57
**DWI**	SVM	0.61	(0.43, 0.78)	0.47	0.78
**DWI**	RF	0.70	(0.54, 0.86)	0.79	0.61

© 2026 The Author(s). Published by Bentham Science Publisher.

This is an open access article distributed under the terms of the Creative Commons Attribution 4.0 International Public License (CC-BY 4.0), a copy of which is available at: https://creativecommons.org/licenses/by/4.0/legalcode. This license permits unrestricted use, distribution, and reproduction in any medium, provided the original author and source are credited.

HOW TO CITE:
Singh A, Shi S, Liu H, Wu Y, Wang Y, Xie J, Li X. Comparison of Radiomic Features from Different MRI Sequences for Predicting Synchronous Liver Metastases after Rectal Cancer. Curr Med Imaging, 2026; 22: e15734056399652.
http://dx.doi.org/10.2174/0115734056399652251029204023
